# Hæmophilia

**Published:** 1902-01-15

**Authors:** S. D. Ruggles

**Affiliations:** Portsmouth, O.


### HÆMOPHILIA.

BY S. D. RUGGLES, D.D.S., PORTSMOUTH, O.

Read at Thirty-sixth Annual Meeting of Ohio Dental Society, December 3, 1901.

A hæmorrhagic diathesis is characterized chiefly by a tendency to spontaneous bleeding from some trivial injury, and is usually accompanied by rheumatoid swelling and pains in the joints.

This disease was known to the ancient writers, but our first reliable information dates from the latter part of the seventeenth century. Concerning its etiology authorities agree that it is always hereditary, cases having been traced through seven generations. The frequency in families varies considerably. While the females of a family are unaffected by the disease they transmit it to their male offspring. This is the rule with few exceptions. Occasionally, however, the transmission may be direct from father to son, grandson, etc. Cases are on record where the family history gave no account of the disease, but the marriages of blood relations have been known to develop hæmophilia in the offspring.

Eighty-six percent of the cases reported are found in males, but in all probability many cases in females are designated by other terms. The disease manifests itself in early life in many cases, but it is seldom that we are called until the removal of permanent teeth is necessary. It occurs in all parts of the world, but more often in the northern countries, especially on sea coasts and in changeable climates. The seasons of the year sometimes affect the frequency of the attacks. This is very noticeable in cold and damp weather.

Upon examination the vessel walls are found to be unusually thin with their lumen considerably altered. The intema is often found to have suffered from fatty degeneration; also the cardiac muscles. The appearance of the blood presents no characteristic features, except that its coagulability is decreased. After prolonged hæmorrhage the blood becomes thin or hydræmic and of a lighter color. Patients suffering from this disease bear the loss of blood more readily than those in perfect health.

The real nature of hæmophilia remains obscure. Coagulation is reduced in the infectious diseases and in leukemia, but records show that these diseases seldom accompany the excessive hæmorrhages considered here. In all probability the florin is affected by chemical changes that are not discernable by our present methods. Coagulation takes place but very slowly. Schmidt claims that fibrin is produced by the interaction of two proteid bodies; viz., fibrinogen and paroglobulin, brought about by the action of some special fibrin ferment, and that when coagulation does not take place it is due to the lack of florinogen. On the other hand,

Hammerstein claims that paroglobulin is not essential. It is generally conceded, however, that a small amount of salts, especially sodium, is necessary for coagulation; but if in excess, coagulation may be prevented altogether. Some authors hold that the vaso-motor system is largely responsible for the conditions in hæmophilia. The hæmorrhage is truly a parenchymatous one, rarely is a larger vessel directly implicated. In fact, deep wounds are more readily controlled in these subjects than superficial ones. Certain parts of the anatomy are more prone than others, mucous membranes especially. In typical cases the joints become swollen and painful, the condition depending upon the hæmorrhage into the capsule and synovial membranes. This extravasation is quite noticeable and in later stages is accompanied by anklyosis.

A correct diagnosis can not be made from the appearance of the patient, for this disease is in no way distinctive, in spite of the detailed descriptions in some of our texts of the *habitus hœmophilicus*. Physical examinations often reveal enlargement of the heart, and systolic murmurs may be heard at the base. The family history together with the symptoms enumerated, should be our guide in a diagnosis.

After the first appearance of the disease the tendency continues throughout life, but when the hæmorrhage is slight it often decreases in after years, some subjects living to a ripe old age. The prognosis is worse for the male.

Prophylaxis is difficult to follow in affected individuals. Every safeguard should be thrown about them, and even the slightest operations as circumcision, extractions, or vaccinations must be avoided, except in the most urgent cases. Marriage should be prevented if possible, especially of the female members.

Immediately upon the occurence of the hæmorrhage the patient should be put at absolute rest. Occasionally this will suffice to allay the trouble, but the application of ice and the various hæmostatic remedies should not be delayed. The more powerful styptics have the disadvantage of forming small clots which in their subsequent separations lead to more serious hæmorrhage than the original. Many remedies are used internally, as perchlorid of iron, ergot, gallic acid and aromatic sulphuric acid. They seem to have little effect in some cases. Calcium chlorid and lime water are said to be more efficient. The effective remedies in our hands were pyrozone, three percent, subsulphate of iron (Monsel's solution) adrenalin and compression.

One of the most obstinate experiences of this sort was with a male about twenty-four years of age, presenting a typical case in every way. The rheumatoid joints had made him an invalid from childhood, and the history of former experiences did not place the surgeon in an enviable position. The cause of this attack was the fracture of a lower second molar during mastication, causing a slight laceration of the gum. Almost a week had passed before a determined effort was made to stop the hæmorrhage. At a consultation with his physician it was decided that extraction was absolutely necessary, for the constant motion of the tongue against the loose piece simply aggravated matters. The first molar was removed at the same time on account of its bad condition. No anesthetics were used. The alveoli were thoroughly washed with three percent pyrozone, and the hæmorrhage ceased in a short time. A pledget of cottonoid the size of the wound was thoroughly dusted with tannic acid and placed in position, on the top of which was placed a guttapercha compress, separating all the teeth about one-quarter of an inch to allow for nourishment or vomiting. A four-inch roller bandage secured the position. Six hours later the patient returned with a profuse hæmorrhage, the operation was repeated and the patient was instructed to keep off his feet and remain as quiet as possible. At two o'clock the following morning I was called to the office by a second return of the trouble which was most obstinate. After removing all the clots and washing with pyrozone the usual arrest of flow did not occur and the entire field remained obscured. A pledget of cotton corresponding in size and shape to each root, was saturated in Monsell's solution and placed in the alveoli. Then a piece of cottonoid saturated in the same and dusted in the powder was placed over all and the compress readjusted. The patient was considerably distressed from the loss of blood, being nauseated and complaining of a severe headache. By daylight the hæmorrhage was reduced sufficiently to remove him to his room. It was now evident that the patient must have absolute quiet and rest. A constant dribbling was kept up for several days in spite of the combined efforts of two physicians and myself. Several remedies were used and as much pressure as we deemed advisable. Having read of a new hæmostatic, supra renal extract, from the kidney of the sheep, we tried it. Fifteen grains in three drachms of boracic acid solution was used first. The hæmorrhage stopped within five minutes and the tissues assumed a blanched appearance. The tissues were not lacerated and no loose pieces of process could be detected. The compress was replaced with a feeling of great relief. For the first time in days had the patient been able to take nourishment without the admixture of blood. A horizontal position was maintained during all this time. At the expiration of five or six hours the hæmorrhage returned. A fresh solution was prepared and the extract applied again with good results. This was continued as often as necessary until the thirteenth day when the hæmorrhage ceased. During this period the patient experienced considerable distress from nausea and headaches. By keeping the alimentary tract open and administering tonics he kept up very well on milk and other liquid foods. A week elapsed before the compress was removed. The wound was entirely closed, the tissues presenting a very delicate pink tint. Antiseptics were used constantly from the beginning.

A few words concerning this remedy may be of interest. Supra renal extract is the active principle of the supra renal glands, is an astringent and hæmostatic of great potency. The aqueous solutions which were used in the above case, gave good results but like all the animal extracts deteriorated rapidly. It is non-irritating, non-toxic, and its hæmostatic properties are due to the contracting of the muscular fibres of the vessel walls. A more staple article is now prepared by Park, Davis & Co., and known as adrenalin. When injected this is a most powerful cardiac stimulant, but its chief use to us is as a hæmostatic. Solutions of 1-10,000 will blanch the normal conjunctiva in less than a minute, while solutions of 1-5,000 are especially serviceable on the gums during the preparation of roots for crowns. It serves a wide field of usefulness in dentistry, eye, ear, nose and throat work, and with no deleterious after-effects.

Bibliography:—Twentieth Century Medicine,

American System of Dentistry, Kirk's Physiology, Wood's Therapeutics.

DISCUSSION.

Dr. Otto Arnold:—The subject presented to us is very important and ought to be thoroughly understood by every member of the dental profession. Serious hæmorrhages occur only in a small percent of cases, but when they do occur then we should be competent to manage such cases in a thoroughly scientific manner. Sometimes unusual complications follow operations for which the dentist may be severely criticized, but these are usually due to inadequate knowledge of existing conditions. Many times these cases pass beyond the control and knowledge of the dentist, in such cases the physician should certainly be consulted. A lack of such knowledge only emphasizes the fact that the modern dentist should be a man of broad attainments, and one capable of representing a profession whose achievements are not confined to technical manipulations only. I am glad this subject has been presented to this body and trust a thorough and intelligent discussion may follow.

The essayist failed to mention the internal administration of any astringent. I have always used gallic acid in ten grain doses, together with local syringing of the socket with hot water, as hot as can be borne, this stimulates the vaso-motor nerves to action, and is preliminary to the use of local astringent remedies. Tannin and supra-renal extract are good astringents. Monsell's solution or any preparation of iron I do not like. They are irritants and secondary hæmorrhage almost invariably follow their use. In reference to horizontal position; gravitation is a factor to be reckoned with, the more the head is elevated the less the flow of blood to that region, so I should advocate putting the patient in a sitting posture. I had a case of a twelve year old girl, a member of a family of bleeders, who had a very serious hæmorrhage once before from the extraction of a temporary tooth. She came to me for an operation and I gave her gallic acid as a prophylactic before commencing. There is no doubt in my mind about this being a systemic condition, so why hesitate to employ preventive systemic remedies in conjunction with local measures.

Dr, J. Taft:—Every member of this body should realize the importance of this subject, we may have at any time a case of this kind. There is perhaps no condition of patients coming to the dentist which is the occasion ot so much anxiety, or which taxes his skill more than these.

The hæmorrhagic diathesis embraces several elements. First, is the structure of the tissues. Often the vascular tissues are very weak in character, they do not have the tenacity and integrity which they should have in a well-balanced normal condition, and are at all times easily ruptured.

Second, there may be an indisposition of the blood to coagulate, maintaining its fluid condition under almost all circumstances, even under any treatment which may be employed for arrest. The blood itself is so changed in character from its normal condition that it fails to coagulate, and though the vessels may not have lost their tone there will be persistent hæmorrhage.

Examine always for abnormal conditions, endeavor to ascertain if there is a physical weakness. Each case will present characteristic manifestations: if a vein is ruptured we get a steady flow from the wound; if the capillaries are ruptured we have an oozing of blood from the injured tissue, and this is not very easily arrested. If an artery is severed we will have an entirely different condition of affairs. The blood will flow in interrupted spurts or pulsations. The color of the blood will also indicate its source; a bright red from the arteries and a darker from veins. So one can always tell from which vessels the hæmorrhage issues and this fact may modify the treatment to be employed. Determine first the blood's coagulability, then the class of vessels from which it occurs; and when these facts are ascertained you have a fairly good basis upon which to begin proper treatment. In treatment reliance is placed upon local applications in too many cases. We are always disposed to employ this. The administration of certain medicaments systemically is in many instances very good indeed. Acetate of lead, opium, gallic acid and a variety of things have been suggested. Where we have constant hæmorrhage the administration of proper systemic medicines may promptly arrest hæmorrhage. In case of abnormal condition of the blood, administer systemically or locally whatever will best increase the coagulability of the blood. Acetate of lead posesses this property. The late Dr. Watt used this material in excessive hæmorrhages, and he claimed for it very decided beneficial properties. His formula was:

℞ Powdered Opium, - grs. jv
Acetate of lead, - - grs. xxiv

Mix and make into eight powders. Take one powder every two hours until the hæmorrhage ceases.

The lead is the astringent agent, while the opium controls the nervousness.—Ed. Register.

Styptics in the cavity or upon bleeding surfaces is a necessary procedure. Many times permanent arrest of hæmorrhage would be accomplished and this will be the more likely in cases where there is a fairly good condition of blood with a predisposition to coagulate, and the more nearly there may be a normal tonic condition of the blood vessels. Under conditions of a full and proper diagnosis cases of hæmorhages need not cause alarm. Sometimes fatal results occur but they are usually in the hands of those utterly incompetent. The management of such conditions involves a greater knowledge of the human system than the average dentist ordinarily possesses, but we should all endeavor to become familiar with these cases and their proper treatment.

Dr. H. A. Smith, of Cincinnati: The ground has been gone over very thoroughly. When the president announced the subject I recalled the past and remembered a long time ago when at every meeting we had some paper on the subject of hæmorrhage after extraction, etc. Have not heard a paper upon this subject since that read by Dr. Arnold, and it is very timely to have another.

Does hæmorrhage occur after extraction of teeth as much as formerly? Cases do not come under my observation as much so as when I was young, neither are cases referred to me so frequently as formerly. I have had most of such cases come to me from other dentists, after night, etc. One of the worst I ever had was referred to me by Dr. Wardle, which I controlled by the use of copper sulphate.

In reference to treatment. There is always a great tendency, and temptation also, to try a variety of styptics. Tannic acid is the only good styptic I have ever used. It is very powerful and combines with the blood producing a comparatively insoluble clot. I believe cases of the worst kind can be cured by the use of this styptic, with pressure for a suitable period, a proper amount of pressure, not too much. Where the bleeding is from the socket it is very easy to control. Hæmorrhages after extraction are mostly from capillaries and arteries. To stop the flow we must apply pressure continuously, which is quite hard to do in this locality. A certain dentist with whom I am acquainted used to stop the flow of blood at the bottom of the socket by the use of a round engine burr lacerating and exposing more tissue upon which the coagulum will adhere. I have sometimes used this expedient, but in most of the cases I make use of applications of tannic acid.

Hæmorrhagic patients are usually anemics, either from this disease or overwork and fatigue. Illustration may be seen in the fatigue resulting in death of animals pursued in a chase. So when these cases present themselves to you if the patient is feeble and an anemic be on your guard, do very little in these cases. We do not proceed in our practice to the extent in extracting teeth as formerly and that is probably the reason we do not see so many cases of hæmorrhage. If the patient is anemic I should hesitate to extract for the blood lacks its coagulability which is its normal property. As a treatment, would endeavor to compress all capillaries, arteries and veins. If I should fail with the use of tannic acid would resort to the use of supra-renal extract.

Dr. Ruggles closing the discussion: The family history of the case which I report is very interesting. One member was blind and had been since birth. This man was a cripple since early infancy. And there was a preacher in the family also, which made it a little worse, other members of the family were more or less afflicted.

In reference to horizontal position all the authorities I looked up on this subject recommended it. In order to supply nourishment to the tissues, increased heart-action was necessary. If you release or reduce uphill tendency of the flow of the blood it will lessen the work of the heart.

While the beat of the heart was high I had to control the nervousness by the use of bromides, etc. I think in this particular case it was a good idea to keep the patient in a horizontal position and as quiet as possible. When I proped him up he became sick at his stomach, and had a headache. We tried internal medication for more than a week, gallic acid in ten grain doses. Iron was also used but this did no good. Previous to this the physician had controlled the hæmorrhage with galic acid, but it did not work this time.

In reference to pressure, I do not advocate extreme pressure, for it will produce irritation and absorption. We kept the bandage tight but did not resort to extreme pressure. Hot water was used also, and used faithfully. I don't care to have another such case. Although the results were satisfactory it caused me many anxious moments. Another practitioner had refused to have anything to do with the case and advised him to go to Cincinnati for treatment.

